# The Action of Reproductive Fluids and Contained Steroids, Prostaglandins, and Zn^2+^ on CatSper Ca^2+^ Channels in Human Sperm

**DOI:** 10.3389/fcell.2021.699554

**Published:** 2021-07-26

**Authors:** Janice K. Jeschke, Cristina Biagioni, Tobias Schierling, Isabel Viola Wagner, Frederik Börgel, Dirk Schepmann, Andreas Schüring, Alexandra E. Kulle, Paul Martin Holterhus, Michael von Wolff, Bernhard Wünsch, Verena Nordhoff, Timo Strünker, Christoph Brenker

**Affiliations:** ^1^Centre of Reproductive Medicine and Andrology, University Hospital Münster, University of Münster, Münster, Germany; ^2^Institute of Pharmaceutical and Medicinal Chemistry, University of Münster, Münster, Germany; ^3^Department of Pediatrics, University Hospital Lübeck, University of Lübeck, Lübeck, Germany; ^4^UKM Kinderwunschzentrum, University Hospital Münster, Münster, Germany; ^5^Division of Pediatric Endocrinology and Diabetes, Department of Pediatrics, Christian-Albrechts-University, Kiel, Germany; ^6^Division of Gynecological Endocrinology and Reproductive Medicine, University Women’s Hospital, Bern, Switzerland; ^7^Cells in Motion Interfaculty Centre, University of Münster, Münster, Germany

**Keywords:** CatSper, human sperm, steroids, prostaglandins, reproductive fluids

## Abstract

The sperm-specific Ca^2+^ channel CatSper registers chemical cues that assist human sperm to fertilize the egg. Prime examples are progesterone and prostaglandin E_1_ that activate CatSper without involving classical nuclear and G protein-coupled receptors, respectively. Here, we study the action of seminal and follicular fluid as well of the contained individual prostaglandins and steroids on the intracellular Ca^2+^ concentration of sperm from donors and *CATSPER2*-deficient patients that lack functional CatSper channels. We show that any of the reproductive steroids and prostaglandins evokes a rapid Ca^2+^ increase that invariably rests on Ca^2+^ influx *via* CatSper. The hormones compete for the same steroid- and prostaglandin-binding site to activate the channel, respectively. Analysis of the hormones’ structure–activity relationship highlights their unique pharmacology in sperm and the chemical features determining their effective properties. Finally, we show that Zn^2+^ suppresses the action of steroids and prostaglandins on CatSper, which might prevent premature prostaglandin activation of CatSper in the ejaculate, aiding sperm to escape from the ejaculate into the female genital tract. Altogether, our findings reinforce that human CatSper serves as a promiscuous chemosensor that enables sperm to probe the varying hormonal microenvironment prevailing at different stages during their journey across the female genital tract.

## Introduction

During their journey to the egg, mammalian sperm face an ever-changing chemical milieu, ranging from seminal fluid over secretions of cells lining the female genital tract and surrounding the egg to follicular fluid, which enters the oviduct upon ovulation. In human sperm, the sperm-specific Ca^2+^ channel CatSper translates changes in the chemical microenvironment into changes of the intracellular Ca^2+^ concentration ([Ca^2+^]_i_) and swimming behavior (Tamburrino et al., 2014; Brown et al., 2019; Wang et al., 2021). Aberrations of *CATSPER* genes, some of which were shown to result in a loss of CatSper function, are associated with male infertility (Avidan et al., 2003; Avenarius et al., 2009; Hildebrand et al., 2010; Smith et al., 2013; Jaiswal et al., 2014; Williams et al., 2015; Brown et al., 2018; Luo et al., 2019; Schiffer et al., 2020; Wang et al., 2020), indicating that CatSper serves as a central signaling knot in human sperm. Quite generally, CatSper is gated by membrane depolarization and intracellular alkalization (Kirichok et al., 2006; Lishko et al., 2010; Seifert et al., 2015; Hwang et al., 2019). In human sperm, CatSper is also activated by prostaglandins and steroids (Lishko et al., 2011; Strünker et al., 2011), the predominant hormones in seminal and follicular fluid (e.g., Bygdeman and Samuelsson, 1966; de los Santos et al., 2012), respectively, which are also released by the oviductal epithelium and cumulus cells surrounding the egg (Ogra et al., 1974; Vastik-Fernandez et al., 1975; Schuetz and Dubin, 1981; Libersky and Boatman, 1995). Our knowledge about the steroid and prostaglandin control of CatSper and human sperm originates especially from studies using progesterone and prostaglandin E (e.g., PGE_1_). Progesterone or PGE_1_ activation of CatSper evokes a rapid Ca^2+^ increase (Blackmore et al., 1990; Baldi et al., 1991; Schaefer et al., 1998; Shimizu et al., 1998; Harper et al., 2003; Bedu-Addo et al., 2007; Strünker et al., 2011; Servin-Vences et al., 2012; Brenker et al., 2018b), which has been implicated in human sperm chemotaxis, hyperactivation, penetration into viscous medium, and/or acrosomal exocytosis (Aitken and Kelly, 1985; Schaefer et al., 1998; Harper et al., 2003; Bedu-Addo et al., 2007; Oren-Benaroya et al., 2008; Publicover et al., 2008; Baldi et al., 2009; Kilic et al., 2009; Servin-Vences et al., 2012; Alasmari et al., 2013; Sanchez-Cardenas et al., 2014; Schiffer et al., 2014; Tamburrino et al., 2014, 2015, 2020; Brown et al., 2017; Rennhack et al., 2018). However, reproductive fluids comprise rather a complex cocktail of various prostaglandins and steroids (Samuelsson, 1963; Bendvold et al., 1987; Munuce et al., 2006; Kushnir et al., 2009; Marchiani et al., 2020). Several steroids identified in follicular fluid evoke Ca^2+^ and motility responses in human sperm that are similar to those evoked by progesterone (Blackmore et al., 1990, 1996; Alexander et al., 1996; Luconi et al., 2004; Brenker et al., 2018b). Only for some of these steroids, e.g., 17-OH-progesterone and estradiol, it has been shown that they also act *via* CatSper (Lishko et al., 2011; Strünker et al., 2011; Brenker et al., 2018a, b; Rehfeld, 2020). Similarly, it has been shown that CatSper is also activated by prostaglandins other than PGEs, such as PGFs, which are also found in seminal fluid (Lishko et al., 2011; Brenker et al., 2018a). However, thus far, a comprehensive quantitative analysis of the action of physiologically relevant prostaglandins and steroids contained in reproductive fluids on [Ca^2+^]_i_ and CatSper in human sperm has been lacking.

Here, we show that stimulation of sperm with follicular and seminal fluid evokes a rapid Ca^2+^ increase that rests predominantly on steroid and prostaglandin activation of CatSper, respectively. Moreover, we determined in a quantitative fashion the steroid content of follicular fluid and studied the action of the steroids on [Ca^2+^]_i_ of human sperm. Similarly, we studied the action of the prostaglandins contained in seminal fluid. Each reproductive steroid and prostaglandin tested, i.e., 15 per class of hormone, evokes Ca^2+^ influx *via* CatSper, yet with different potency and efficacy; in sperm from *CATSPER2*-deficient patients that lack functional CatSper channels, the hormone-induced Ca^2+^ influx is abolished. Analysis of the structure–activity relationship revealed the particular structural features rendering a hormone more or less potent to activate the channel. Furthermore, we show that all reproductive steroids employ the same steroid-binding site and, thereby, compete for CatSper activation, whereas prostaglandins compete all for the same prostaglandin-binding site to activate the channel. Altogether, our results suggest that the hormonal control of CatSper and, thereby, sperm function involves a concerted (inter)action of the various prostaglandins and steroids that sperm are exposed to within the female genital tract. Finally, we show that Zn^2+^, which is present at millimolar concentration in seminal fluid, suppresses hormone activation of CatSper. The complex opposing action of seminal Zn^2+^ and prostaglandins on CatSper might prevent premature activation of CatSper in the ejaculate and serve as a dilution sensor for sperm, assisting sperm to escape from the ejaculate into the female genital tract.

## Results

### Follicular and Seminal Fluid-Evoked Ca^2+^ Signals in Human Sperm Are Predominantly Mediated by CatSper

**FIGURE 1:**
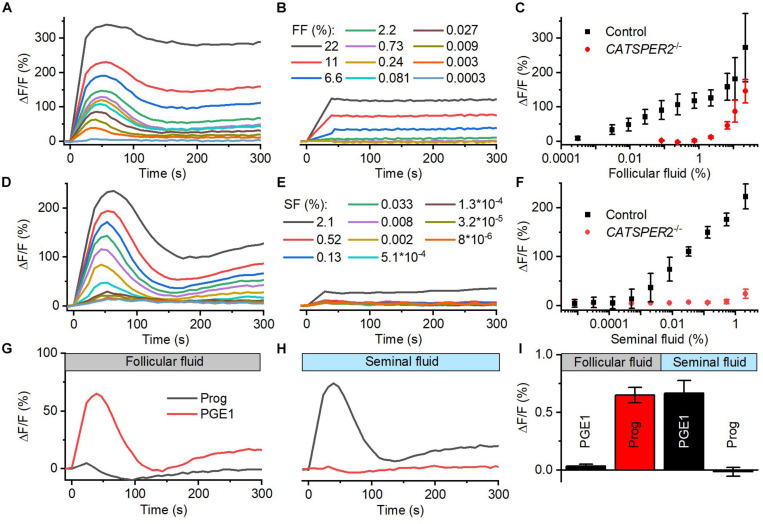
Follicular and seminal fluid-evoked Ca^2+^ signals in human sperm. **(A)** Representative Ca^2+^ signals evoked by dilute solutions of follicular fluid (FF); the final FF concentration is indicated as volume/volume percentage. [Ca^2+^]_i_ was monitored in control sperm from donors loaded with a fluorescent Ca^2+^ indicator, using a fluorescence plate reader. ΔF/F (%) indicates the percent change in fluorescence (ΔF) with respect to the mean basal fluorescence (F) before application (and subsequent continuous presence) of FF at *t* = 0. **(B)** Representative FF-evoked Ca^2+^ signals in sperm from patients deficient for the *CATSPER2* gene (*CATSPER2*^–/–^), lacking functional CatSper channels. **(C)** Concentration–response relationship for the mean (±SD) maximal signal amplitudes evoked by FF in control and CATSPER2^–/–^ sperm (*n* = 3). **(D)** Representative Ca^2+^ signals evoked by dilute solutions of seminal fluid (SF) in control **(D)** and *CATSPER2*^–/–^ **(E)** sperm. **(F)** Concentration–response relationship for the mean (±SD) maximal signal amplitudes (*n* = 3). **(G)** Representative progesterone- and PGE_1_-evoked Ca^2+^ signal in control sperm preincubated in 1% FF or **(H)** 0.5% SF; progesterone or PGE_1_ was applied at *t* = 0. **(I)** Mean (±SD) maximal amplitude of progesterone- and PGE_1_-evoked Ca^2+^ signals in control sperm preincubated in 1% FF or 0.5% SF (*n* = 4).

We studied the action of follicular (FF) and seminal fluid (SF) on the intracellular Ca^2+^ concentration of human sperm. To this end, populations of human sperm were challenged with dilute solutions of the respective fluid and [Ca^2+^]_i_ was monitored using a fluorescent Ca^2+^ indicator. In sperm from donors, both FF and SF evoked a rapid [Ca^2+^]_i_ increase, consisting of a Ca^2+^ transient (Figures 1A–F) followed by a second sustained elevation of [Ca^2+^]_i_ (Figures 1A,D); similar FF-induced Ca^2+^ signals were reported before (Thomas and Meizel, 1988; Blackmore et al., 1990; Hong et al., 1993; Brown et al., 2017). The amplitude of the Ca^2+^ signal increased in a dose-dependent fashion: the FF-induced signal first saturated at about 1% FF (final concentration, volume/volume percentage), but then continued to rise at FF ≥ 2.2% (Figures 1A,C), whereas the signal evoked by SF increased rather steadily with increasing SF (Figures 1D,F). It has been suggested that FF-induced Ca^2+^ signals rest predominantly, but not exclusively, on Ca^2+^ influx *via* CatSper (Brown et al., 2017). Supporting this notion and indicating that CatSper also mediates the signals induced by SF, Ca^2+^ signals evoked by ≤2.2% FF and <2% SF were abolished in sperm from *CATSPER2*-deficient patients (Figures 1B,E) lacking functional CatSper channels (see Brenker et al., 2018b; Schiffer et al., 2020; Wang et al., 2020); at higher concentrations, both FF and SF evoked a residual, sustained [Ca^2+^]_i_ increase in *CATSPER2^–/–^* sperm that might rest on inhibition of Ca^2+^ export and/or Ca^2+^ release from intracellular stores. We can, however, not exclude that the residual signal reflects an artifactual increase in the fluorescence of indicators rather than a genuine [Ca^2+^]_i_ response.

The activation of CatSper by reproductive fluids does not rest on an increase of the intracellular pH (pH_i_): SF did not affect pH_i_ of human sperm (Supplementary Figures 1C,D), and FF slightly decreased pH_i_ (Supplementary Figures 1A,B), yet only at high concentrations. Cross-desensitization experiments demonstrate that the activation of CatSper by FF and SF is rather mediated by the contained steroids and prostaglandins, respectively: In sperm preincubated in FF, progesterone- but not PGE_1_-evoked Ca^2+^ influx was abolished (Figures 1G,I), whereas preincubation of sperm in SF abolished PGE_1_- but not progesterone-induced Ca^2+^ influx (Figures 1H,I). We investigated the action of the individual steroids and prostaglandins in the reproductive fluids in more detail.

### Analysis of the Steroid Content of Human Follicular Fluid

We determined by LC-MS/MS the concentration of 16 steroids, representing all major classes, in follicular fluid obtained during ovum pickup for assisted reproduction from gonadotropin-stimulated patients; FF of eight ova from four patients was pooled to yield averaged concentrations. The FF contained only picomolar concentrations of deoxycortisols, whereas all other steroids were present at nano- to micromolar concentrations (Table 1). These results are in line with those of previous studies (e.g., Walters et al., 2019). The steroid concentrations in pooled FF from 10 ova obtained from 10 patients not stimulated with gonadotropin are shown in Supplementary Table 1.

**TABLE 1:** Steroid composition of pooled human follicular fluid.

Concentration of steroids in FF (nM)			
Progesterone	28,880	Cortisone	20
Pregnenolone	1,470	Androstendione	4
17-OH-progesterone	2,982	Androstenediol	20
17-OH-pregnenolone	115	Dehydroepiandrosterone	463
11-Deoxycorticosterone	65	Testosterone	2
Corticosterone	6	Estradiol	1,221
11-Deoxycortisol	0.03	Estrone	76
21-Deoxycortisol	0.19	Estriol	8

### The Action of Reproductive Steroids on CatSper in Human Sperm

**FIGURE 2:**
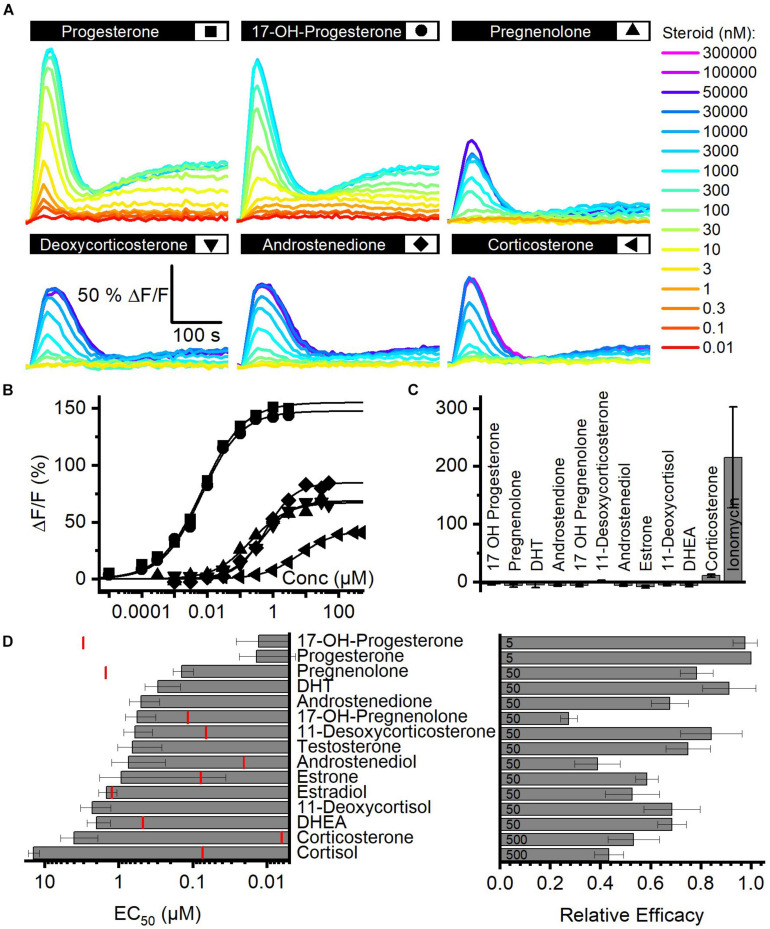
Steroid-evoked Ca^2+^ signals in human sperm. **(A)** Representative Ca^2+^ signals evoked by progesterone, 17-OH-progesterone, pregnenolone, deoxycorticosterone, corticosterone, or androstenedione in control sperm from donors. **(B)** Concentration–response relationship for the maximal amplitudes of signals shown in **(A)**. **(C)** Mean (±SD) maximal amplitude of Ca^2+^ signals evoked by steroids [at the concentration indicated in **(D)**; right panel] or the Ca^2+^-ionophore ionomycin (2 μM) in *CATSPER2^–/–^* sperm (*n* = 3). **(D)** Left: potency, i.e., mean (±SD) EC_50_ (*n* ≥ 4), of steroids to evoke Ca^2+^ signals in control sperm (red lines indicate the concentration of the steroid in follicular fluid); right: relative efficacy of steroids to evoke Ca^2+^ signals in control sperm, i.e., mean (±SD) maximal signal amplitude evoked by the respective steroid at the indicated saturating concentration (in μM) relative to that evoked by progesterone (set to 1) (*n* ≥ 5).

We determined the structure–activity relationship for the activation of human CatSper by steroids contained in FF. To this end, we studied Ca^2+^ signals evoked by individual steroids and analyzed their concentration–response relationship (Figure 2A). With no exception, each of the 15 steroids tested evoked a biphasic Ca^2+^ response (Figures 2A,B,D) that was abolished in *CATSPER2^–/–^* sperm (Figure 2C; Brenker et al., 2018b), demonstrating that the response rests on Ca^2+^ influx *via* CatSper. The steroids differed, however, in their potency and efficacy to evoke Ca^2+^ influx (Figures 2B,D and Supplementary Table 2) (Brenker et al., 2018b). With decreasing potency, also the efficacy tended to decrease, indicating that the two parameters are correlated (Figure 2D and Supplementary Figure 2).

Moreover, we recognized that patterns of pharmacological properties coincide with structural features. First, progesterone and 17-OH-progesterone represent the most potent and efficacious steroids and activate CatSper with similar potency and efficacy. This indicates that the introduction of an OH group at the 17α-position of progesterone does not affect the activation of the channel. Modifications of progesterone and 17-OH-progesterone at the same positions affect the potency in a similar fashion: reduction of the ketone at 3-position and a shift of the double bond from 4/5- to 5/6-position decreases the potency by about 10-fold (Figure 2D; compare pregnenolone and 17-OH-pregnenolone to progesterone and 17-OH-progesterone, respectively). Increasing the polarity by the successive addition of OH groups at 21-position (deoxycorticosterone) and 17α- as well as 21-position (11-deoxycortisol) decreases the potency by 10- and 100-fold, respectively. The potency to activate CatSper also decreases with the number of OH groups attached to the cholesterol backbone, though OH groups attached to the 17α-position are better tolerated than groups attached to 11-position; relative potency: cortisol (OH at C-11, C-17α, and C-21) < corticosterone (OH at C-11 and C-21) < 11-deoxycortisol (OH at C 17α and C-21) < deoxycorticosterone (OH at C-21). Finally, steroids lacking an acyl group at 17-position (androgens) are 100-fold less potent than progesterone, whereas the presence of an OH (Figure 2D, compare estrone versus testosterone and androstenediol) or a keto group (Figure 2D; compare dehydroepiandrosterone versus androstenedione and estradiol) at 17-position hardly affects the potency. The group at 3-position does not affect the potency either: compare χ,δ-unsaturated alcohol (dehydroepinadrosterone versus androstenediol), α,β-unsaturated ketone (androstenedione testosterone), and phenol (estrone, estradiol). In a nutshell, augmenting the steroid polarity by additional OH groups hampers CatSper activation, especially for the glucocorticoids cortisol and corticosterone, where the OH group is bound to C11 of the cholesterol backbone.

### Reproductive Steroids Compete for the Same Binding Site to Activate CatSper

**FIGURE 3:**
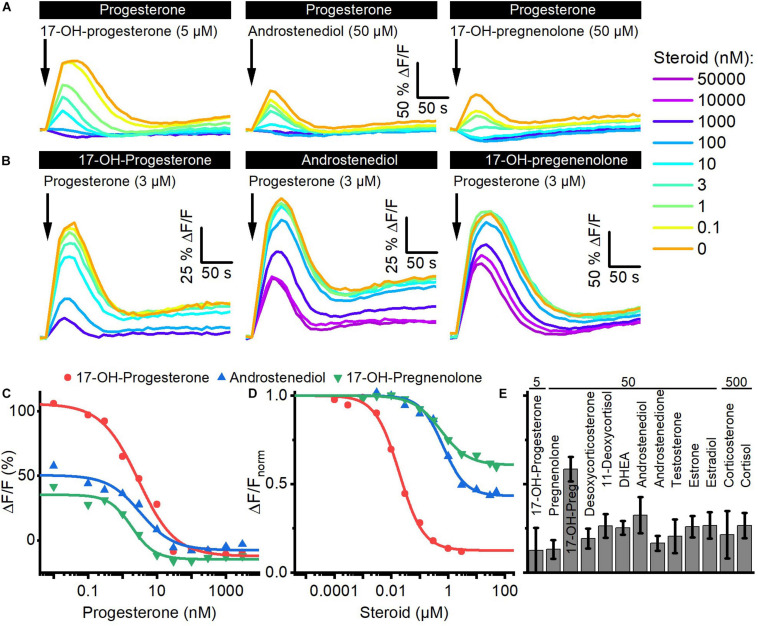
Steroids compete for the same binding site to activate CatSper in human sperm. **(A)** Representative Ca^2+^ signals evoked by a saturating concentration of 17-OH-progesterone (5 μM), androstenediol (50 μM), or 17-OH-pregnenolone (50 μM) in sperm preincubated in different concentrations of progesterone. **(B)** Representative Ca^2+^ signals evoked by a saturating concentration of progesterone (3 μM) in sperm preincubated in different concentrations of 17-OH-progesterone, androstenediol, or 17-OH-pregnenolone. **(C)** Maximal amplitude of steroid-evoked Ca^2+^ responses shown in **(A)**, plotted as a function of the (preincubated) progesterone concentration. **(D)** Maximal amplitude of the progesterone-evoked Ca^2+^ responses shown in **(B)**, plotted as a function of the (preincubated) steroid concentration. **(E)** Mean (±SD) maximal amplitude of the Ca^2+^ signal evoked by progesterone (3 μM) in sperm preincubated in the indicated (in μM) saturating concentration of the respective steroid (*n* = 3–8).

We investigated whether the steroids in follicular fluid employ the same binding site to activate CatSper. To this end, we performed cross-desensitization experiments (Strünker et al., 2011; Brenker et al., 2018b): we challenged sperm preincubated in different concentrations of progesterone with a saturating concentration of 17-OH-progesterone, androstenediol, or 17-OH-pregnenolone and monitored the ensuing Ca^2+^ response (Figures 3A,C). Progesterone suppressed in a concentration-dependent fashion the steroid-evoked Ca^2+^ signals; at > 300 nM progesterone, i.e., concentrations at which the amplitude of the Ca^2+^ signal evoked by progesterone itself saturate (Figure 2B), the 17-OH- progesterone-, androstenediol-, or 17-OH-pregnenolone-evoked signals were abolished (Figures 3A,C). The IC_50_ of progesterone to suppress the steroid responses was 8.5 ± 6.1 nM for 17-OH-progesterone, 5.0 ± 2.8 nM for androstenediol, and 2.6 ± 0.9 nM for 17-OH-pregnenolone (*n* = 3) (Figure 3C), which is similar to the EC_50_ of progesterone to evoke Ca^2+^ signals in human sperm (Figure 2B). *Vice versa*, we challenged sperm preincubated in different concentrations of 17-OH-progesterone, androstenediol, or 17-OH-pregnenolone with a saturation concentration of progesterone and monitored the ensuing Ca^2+^ signal (Figures 3B,D). The progesterone-evoked Ca^2+^ signal was suppressed by 17-OH-progesterone, androstenediol, and 17-OH-pregnenolone in a concentration-dependent fashion (Figures 3B,D) with an IC_50_ of 23.8 ± 7.27 nM (*n* = 4), 2.5 ± 1.7 μM (*n* = 3), and 1.63 ± 2.33 μM (*n* = 3), respectively, which is similar to the EC_50_ value of the respective steroid to evoke Ca^2+^ signals (Figure 2D). Of note, the progesterone response was completely abolished by preincubation with 17-OH-progesterone, but not with androstenediol or 17-OH-pregnenolone (Figures 3B,D). For example, upon preincubation with ≥ 10 μM androstenediol or 17-OH-pregnenolone, the amplitude of the progesterone-evoked Ca^2+^ response settled at a constant residual level. Residual progesterone-induced Ca^2+^ signals were also observed upon preincubation with a saturating concentration of any of the other steroids identified in follicular fluid (Figure 3E). Together, these results demonstrate that the reproductive steroids compete all for the same binding site to activate CatSper. Moreover, displacing a weakly (e.g., androstenediol, 17-OH-pregnenolone) with a highly efficacious steroid (e.g., progesterone) from the steroid-binding site reinduces Ca^2+^ influx *via* CatSper; the amplitude of the Ca^2+^ signal correlates negatively with the efficacy of the steroid displaced (Figures 2D, 3D).

### The Action of Reproductive Prostaglandins on CatSper in Human Sperm

**FIGURE 4:**
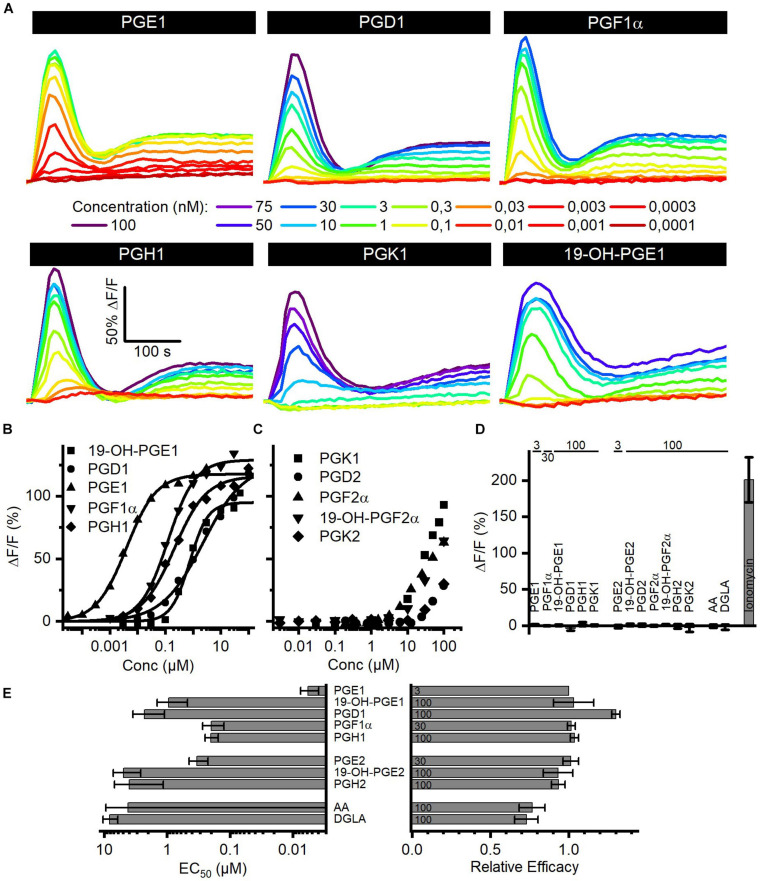
Prostaglandin-evoked Ca^2+^ signals in human sperm. **(A)** Representative Ca^2+^ signals evoked by PGE_1_, PGD_1_, PGF_1α_, PGH_1_, or 19-OH-PGE_1_ in control sperm from donors. **(B)** Concentration–response relationship for the maximal amplitudes of Ca^2+^ signals shown in **(A)**, except for PGK_1_. **(C)** Concentration–response relationship for the maximal amplitudes of Ca^2+^ signals evoked by PGK_1_, PGD_2_, PGF_2α_, 19-OH-PGF_2α_, or PGK_2_; the concentration–response relationships could not be fitted to derive an EC_50_, because the signal amplitudes did not saturate at the highest concentration tested. **(D)** Mean (±SD) maximal amplitude of Ca^2+^ signals evoked by prostaglandins (at the concentration indicated in μM) or the Ca^2+^-ionophore ionomycin (2 μM) in *CATSPER2^–/–^* sperm (*n* = 3–5). **(E)** Left: potency, i.e., mean (±SD) EC_50_ (*n* = 3–4), of prostaglandins to evoke Ca^2+^ signals in control sperm; right: relative efficacy of prostaglandins to evoke Ca^2+^ signals in control sperm, i.e., mean (±SD) maximal signal amplitude evoked by the respective prostaglandin at the indicated saturating concentration (in μM) relative to that evoked by PGE_1_ (set to 1) (*n* = 3–4).

Seminal fluid contains a cocktail of various prostaglandins at concentrations from the low nano- to the high micromolar range (Table 2). We determined the structure–activity relationship for the activation of human CatSper by reproductive prostaglandins as well as prostaglandin H_1_ and H_2_ (PGH_1_, PGH_2_) and the prostaglandin precursors arachidonic acid (AA) and dihomo-γ-linolenic acid (DGLA). To this end, we studied Ca^2+^ signals evoked by the individual prostaglandins and analyzed the concentration–response relationships (Figures 4A,B). Each of the prostaglandins tested evoked a biphasic Ca^2+^ signal that was abolished in *CATSPER2^–/–^* sperm (Figures 4A,D), demonstrating that the response rests on Ca^2+^ influx *via* CatSper. The prostaglandins differed considerably in their potency to increase [Ca^2+^]_i_, but in contrast to steroids, the efficacy was rather similar (Figures 4A,E and Supplementary Figure 2) and did not correlate with the potency (compare Figures 2D, 4E and Supplementary Figure 2). Of note, we failed to determine an EC_50_ for PGK_1_, PGK_2_, PGF_2α_, 19-OH-PGF_2α_, and PGD_2_. Even at the highest possible concentration tested, i.e., 100 μM, the amplitude of the Ca^2+^ signal did not reach saturation (Figure 4C), indicating that these prostaglandins activate CatSper with very low potency. PGE_1_ represented the most potent prostaglandin. In general, prostaglandins with one double bond (PGX_1_) are more potent than their counterpart with two double bonds (PGX_2_). For example, PGE_2_ is about 55-fold less potent than PGE_1_. Moreover, PGE_1_ contains a ketone in 9-position and an alcohol in 11-position. Changing the ketone into an alcohol decreases the potency only by 20-fold (Figures 4B,E, compare PGE_1_ versus PGF_1α_), whereas the presence of ketone at 11-position (compare PGE_1_ versus PGD_1_ and PGK_1_) decreases the potency considerably, i.e., by at least 1,000-fold (Figures 4B,E, compare PGE_1_ versus PGD_1_). Remarkably, a ketone at 11-position seems to increase the efficacy (Figure 4B, compare PGE_1_ versus PGD_1_). The finding that a peroxide bicyclic to the cyclopentane decreases the potency by only about 35-fold (Figure 4B, compare PGE_1_ versus PGH_1_) suggests that the OH group at 11-position is beneficial, but not required, for a submicromolar EC_50_. Introduction of an OH group at 19-position decreases the potency, however, considerably (Figure 4B, compare PGE_1_ versus 19-OH-PGE_1_). Finally, both AA and DGLA comprise several double bonds instead of O-atom-containing functional groups. AA and DGLA are about 1,000-fold less potent than PGE_1_. Nevertheless, they are still more potent than some of the prostaglandins tested (e.g., PGK_1_, PGK_2_, PGF_2α_, 19-OH-PGF_2α_, and PGD_2_).

**TABLE 2:** Prostaglandin concentrations in SF.

Concentrations of prostaglandins in SF (μM)	
19-OH-PGE_1+2_	720 ± 369
19-OH-PGF_1α_	58 ± 1
PGE_2_	33 ± 12
PGE_1_	25 ± 15
PGF_1α_	7 ± 4
PGF_2α_	6 ± 2

*Mean (±SD) concentration reported by Bygdeman and Samuelsson (1966); Isidori et al. (1980), Gerozissis et al. (1982); Aitken and Kelly (1985), and Schaefer et al. (1998).*

### Reproductive Prostaglandins Compete for the Same Binding Site to Activate CatSper

**FIGURE 5:**
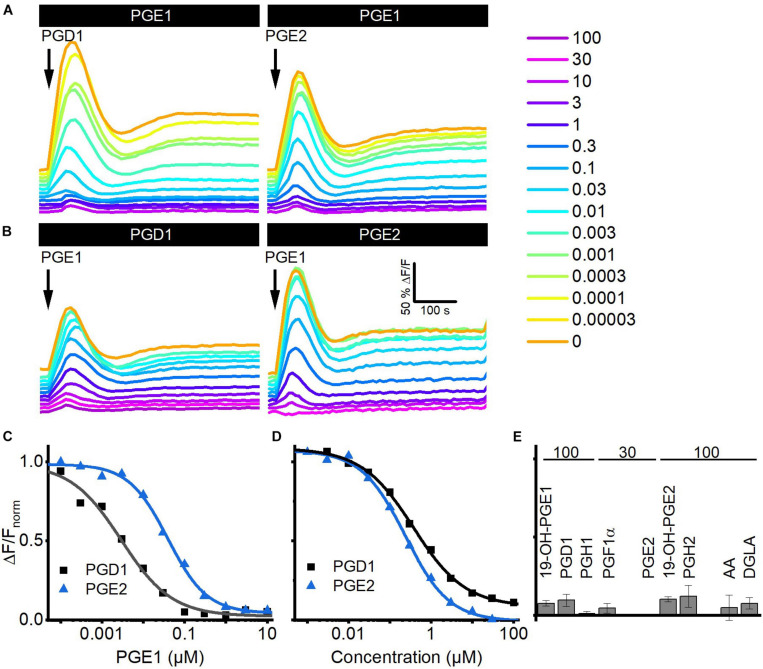
Prostaglandins compete for the same binding site to activate CatSper in human sperm. **(A)** Representative Ca^2+^ signals evoked by a saturating concentration of PGD_1_ (100 μM) and PGE_2_ (30 μM) in sperm preincubated in different concentrations of PGE_1_. **(B)** Representative Ca^2+^ signals evoked by a saturating concentration of PGE_1_ (3 μM) in sperm preincubated in different concentrations of PGD_1_ or PGE_2_. **(C)** Maximal amplitude of PGD_1_- and PGE_2_-evoked Ca^2+^ responses shown in **(A)**, plotted as a function of the (preincubated) PGE_1_ concentration. **(D)** Maximal amplitude of the PGE_1_-evoked Ca^2+^ responses shown in **(B)**, plotted as a function of the (preincubated) PGD_1_ or PGE_2_ concentration. **(E)** Mean (±SD) maximal amplitude of PGE_1_-evoked Ca^2+^ signals in sperm preincubated in the indicated (in μM) saturating concentrations of the respective prostaglandin (*n* = 3–4).

We investigated whether prostaglandins employ the same binding site to activate CatSper using cross-desensitization experiments. We challenged sperm preincubated in different concentrations of PGE_1_ with a saturating concentration of PGD_1_ or PGE_2_. PGE_1_ suppressed in a concentration-dependent fashion the prostaglandin-evoked Ca^2+^ signals; at > 0.1 μM PGE_1_, i.e., concentrations at which the amplitude of Ca^2+^ signals evoked by PGE_1_ itself saturate, the PGD_1_- and PGE_2_-evoked signals were completely abolished (Figures 5A,C). The IC_50_ of PGE_1_ to suppress the prostaglandin responses was 2.2 ± 0.6 nM for PGD_1_ and 18 ± 7 nM for PGE_2_ (*n* = 3), which is similar to the EC_50_ of PGE_1_ to evoke Ca^2+^ signals in human sperm. *Vice versa*, in sperm preincubated in PGD_1_ or PGE_2_, the PGE_1_-induced Ca^2+^ signal was suppressed in a concentration-dependent fashion with an IC_50_ of 0.7 ± 0.4 and 0.24 ± 0.01 μM (*n* = 3) (Figures 5B,D), respectively, which is similar to the EC_50_ of the respective prostaglandin to evoke Ca^2+^ signals in human sperm. Of note, the PGE_1_ response was completely abolished upon preincubation with PGD_1_ and PGE_2_ (Figures 5B,D). Similarly, the PGE_1_-evoked Ca^2+^ signal was almost completely suppressed upon preincubation with a saturating concentration of any other prostaglandin tested (Figure 5E), reflecting the rather similar efficacy among prostaglandins to activate CatSper. Altogether, these results show that reproductive prostaglandins compete for the same prostaglandin-binding site to activate CatSper.

### Zn^2+^ Inhibits Prostaglandin Activation of CatSper

**FIGURE 6:**
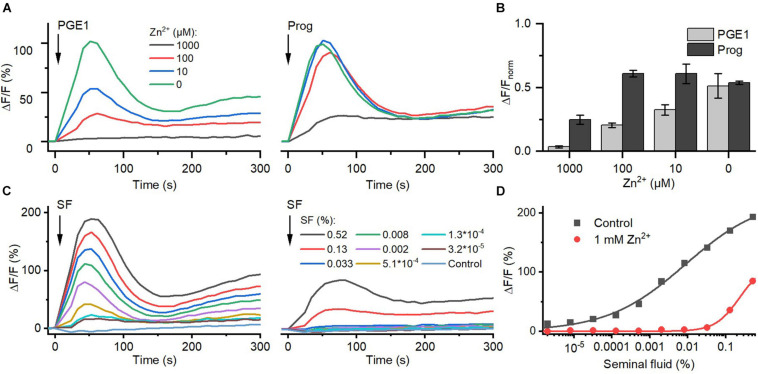
The action of Zn^2+^ on prostaglandin- and steroid-evoked Ca^2+^ signals in human sperm. **(A)** Representative Ca^2+^ signal evoked by progesterone (3 μM) and PGE_1_ (3 μM) in the absence and presence of different Zn^2+^ concentrations. **(B)** Mean (±SD) maximal amplitude of Ca^2+^ signals evoked by progesterone and PGE_1_ in the presence of indicated Zn^2+^ concentration normalized to the amplitude of the ionomycin-evoked Ca^2+^ signal recorded in parallel. **(C)** Representative seminal fluid-evoked Ca^2+^ signals in the absence (left) and presence (right) of Zn^2+^ (1 mM). **(D)** Concentration–response relationship for the maximal amplitudes of Ca^2+^ signals shown in **(B)**.

Seminal fluid contains millimolar concentrations of Zn^2+^ (Cooper et al., 1991; Zhao et al., 2016). We investigated the action of Zn^2+^ on prostaglandin- and steroid-evoked Ca^2+^ influx *via* CatSper. At ≥10 μM, Zn^2+^ suppressed PGE_1_-induced Ca^2+^ influx in a dose-dependent fashion; the signal was almost completely abolished at 1 mM Zn^2+^ (Figures 6A,B). In contrast, at ≤100 μM, Zn^2+^ did not affect progesterone-evoked Ca^2+^ influx, and at 1 mM Zn^2+^, the signal amplitude was reduced only by about 60%. Of note, as a reference, we also recorded Ca^2+^ signals evoked by ionomycin in the absence and presence of Zn^2+^ (Supplementary Figure 3) and corrected for the Zn^2+^-induced decrease in the maximal signal amplitude (Figure 6B). Moreover, in sperm bathed in Zn^2+^ (1 mM), the Ca^2+^ influx evoked by ≤0.03 and >0.1% seminal fluid was also abolished and attenuated, respectively (Figures 6C,D). This indicates that Zn^2+^ suppresses prostaglandin activation of CatSper in the ejaculate.

### The Concerted Action of Prostaglandins and Zn^2+^ on CatSper Might Serve as a Dilution Sensor for Human Sperm

**FIGURE 7:**
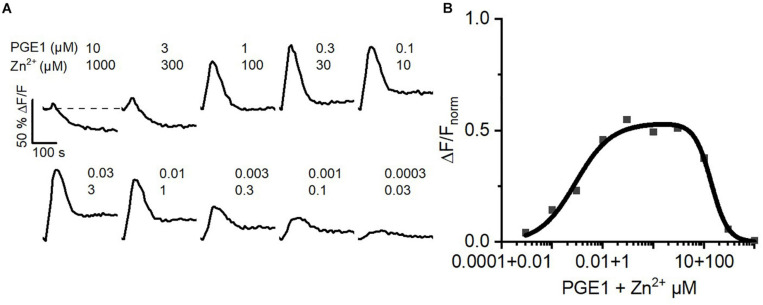
Ca^2+^ signals evoked by dilute solutions of a Zn^2+^/PGE_1_ mixture. **(A)** Representative Ca^2+^ signals in human sperm stimulated with a serially diluted mixture of 10 μM PGE_1_ and 1 mM Zn^2+^. **(B)** Concentration–response relationship for the maximal amplitudes of Ca^2+^ signals shown in **(A)** relative to that evoked by PGE_1_ (3 μM) in the absence of Zn^2+^ (set to 1).

Inside the female genital tract, the ejaculate dilutes with female reproductive fluids and sperm escape from the ejaculate into the uterus and oviduct. Thus, *in vivo*, at the beginning of their journey across the female genital tract, sperm experience a decreasing concentration gradient of both seminal prostaglandins and Zn^2+^. We tried to emulate this scenario *in vitro*. To this end, we challenged sperm with serial dilutions of buffer containing 10 μM PGE_1_ and 1 mM Zn^2+^, i.e., a concentration ratio similar to that in the ejaculate. At low dilutions, the PGE_1_/Zn^2+^ mixture rather decreased [Ca^2+^]_i_, confirming that Zn^2+^ suppresses prostaglandin activation of CatSper (Figure 7A). However, with increasing dilution factor, the Zn^2+^ inhibition tapered off, giving rise to prostaglandin-evoked Ca^2+^ signals. The signal amplitude first grew with increasing dilution factor until it decreased again due to the decreasing PGE_1_, yielding a bell-shaped dilution–response relationship (Figure 7B). Sperm might harness the PGE_1_/Zn^2+^ interplay as a dilution sensor: in the ejaculate, the inhibition of prostaglandin-evoked Ca^2+^ influx by Zn^2+^ prevents premature activation of CatSper. However, upon dilution of the ejaculate, when sperm start escaping it *in vivo*, sperm are relieved from Zn^2+^ inhibition and the prostaglandin action commences. The relief of the Zn^2+^ inhibition and ensuing prostaglandin-evoked motility responses might promote the escape from the ejaculate into the female genital tract.

## Discussion

In the past three decades, work by many groups, including our own, demonstrated that various steroids and prostaglandins increase [Ca^2+^]_i_ in human sperm (e.g., Blackmore et al., 1990; Krausz et al., 1995; Alexander et al., 1996; Blackmore et al., 1996; Schaefer et al., 1998; Luconi et al., 2004; Baldi et al., 2009; Strünker et al., 2011; Brenker et al., 2018a, b; Rehfeld, 2020). Here, using CatSper-deficient sperm from infertile patients lacking the *CATSPER2* gene, we show that Ca^2+^ responses evoked by reproductive steroids and prostaglandins rest invariably on Ca^2+^ influx *via* CatSper.

Our results are in line with that of most (Blackmore et al., 1990, 1996; Luconi et al., 1999, 2001; Rossato et al., 2005; Brenker et al., 2018b; Rehfeld, 2020), but not all, previous studies concerning the steroid action in sperm: Mannowetz et al. (2017) claimed that testosterone, hydrocortisone, and estradiol represent antagonists that abolish steroid activation of CatSper but themselves do not activate the channel. However, our results and those of a number of other, independent studies consistently show that also testosterone, hydrocortisone, and estradiol are, in fact, true agonists that activate CatSper (Luconi et al., 1999, 2001; Rossato et al., 2005; Brenker et al., 2018b; Rehfeld, 2020). This is not to say that CatSper is activated by any steroidal compound: levonorgestrel, the synthetic steroid used in intrauterine contraceptive devices, does not evoke a Ca^2+^ signal (Supplementary Figure 4), supporting the notion that C-17α is a key position for CatSper activation that does not tolerate an alkyne group. The steroid control of CatSper is a prime example for nonclassical steroid action, involving membranous rather than nuclear steroid receptors (Baldi et al., 2009; Tamburrino et al., 2020). It has been proposed that in sperm, steroids activate the membrane serine hydrolase α/β hydrolase domain-containing protein 2 (ABHD2), which in turn degrades 2-AG in the flagellar membrane, relieving CatSper from inhibition by 2-AG (Miller et al., 2016). We show that steroids increase [Ca^2+^]_i_ in sperm with vastly different potency and efficacy (see also Luconi et al., 1999; Brenker et al., 2018b), suggesting that the hormones bind to ABHD2 with different affinities and act as either full or partial agonists. However, the pharmacology of the steroid-binding site supposedly controlling ABHD2 is unknown. Thus, to scrutinize the model of nonclassical steroid action in sperm, it is required to determine whether the pharmacology of the steroid activation of ABHD2 and CatSper matches. Of note, at high micromolar concentrations, progesterone activates not only CatSper, but also inhibits Slo3 (Brenker et al., 2014), the principal K^+^ channel in human sperm (Brenker et al., 2014). Whether steroids other than progesterone also target Slo3 is unknown and whether and how inhibition of Slo3 affects the potency and/or efficacy of steroids to evoke Ca^2+^ influx *via* CatSper remains to be determined.

Moreover, the efficacy to elevate [Ca^2+^]_i_ might co-determine the gamut of behavioral responses evoked by a particular steroid. Not only progesterone but also estradiol, testosterone, and hydrocortisone promote the penetration of sperm into viscous medium, indicating that this behavioral response is rather independent of the efficacy of steroids (Rehfeld, 2020). By contrast, progesterone, but not estradiol, also evokes acrosomal exocytosis (Luconi et al., 2001; Rossato et al., 2005), suggesting that the increase of [Ca^2+^]_i_ by estradiol is not sufficient to trigger acrosomal exocytosis. However, it needs to be systematically assessed whether the efficacy of steroids to increase [Ca^2+^]_i_ and their ability to evoke particular cellular responses indeed correlate. Furthermore, on a single-cell level, progesterone activation of CatSper triggers long-lasting oscillations of [Ca^2+^]_i_ (Kirkman-Brown et al., 2004; Bedu-Addo et al., 2007; Brown et al., 2017; Kelly et al., 2018; Mata-Martinez et al., 2018; Torrezan-Nitao et al., 2021) that seem to involve Ca^2+^-induced Ca^2+^ release and an interplay of CatSper with Slo3 (Torrezan-Nitao et al., 2021). Whether steroids other than progesterone also trigger such [Ca^2+^]_i_ oscillations is unknown. However, the finding that steroid- and prostaglandin-induced Ca^2+^ signals are completely abolished in *CATSPER2^–/–^* sperm demonstrates that the hormones do not directly stimulate Ca^2+^ release.

Another question concerns the physiological significance of the promiscuous steroid activation of CatSper. Figure 2D shows that for some, but not all steroids, the concentration in follicular fluid suffices to activate CatSper. This suggests that *in vivo*, these steroids interplay to control the activity of CatSper. However, upon ovulation, only trace amounts of follicular fluid indeed enter the oviduct (Hansen et al., 1991). This suggest that at physiological dilutions, the steroid activation of CatSper by follicular fluid rests predominantly on the action of progesterone, representing by far the most abundant, efficient, and potent steroid. Supporting this notion, the action of follicular fluid on CatSper and human sperm is largely, but not entirely, mimicked by progesterone (Oren-Benaroya et al., 2008; Brown et al., 2017). Previous studies suggest that so far unknown components of follicular fluid other than steroids also act on CatSper and that follicular fluid increases [Ca^2+^]_*i*_ by mechanisms independent of CatSper (Brown et al., 2017). The latter is supported by our finding that high concentrations of follicular fluid evoke sizeable Ca^2+^ signals even in *CATSPER2^–/–^* sperm. Finally, steroids are not only contained in follicular fluid, but also in the secretions of the cumulus cells and cells lining the oviduct (Libersky and Boatman, 1995; Munuce et al., 2006; Lamy et al., 2016). Similar to follicular fluid, progesterone is the most abundant steroid in the secretion of cumulus cells (Osman et al., 1989), suggesting that also in the vicinity of the egg, steroid activation of CatSper rests predominantly on the action of progesterone. The steroid composition of oviductal fluid is, however, largely unknown in quantitative terms. Thus, at different stages during their journey across the oviduct, steroids other than progesterone might take center stage as to the control of CatSper and, thereby, the swimming behavior of human sperm.

The mechanism of CatSper activation by prostaglandins has remained elusive, except that it does not involve ABHD2, classical G protein-coupled receptors, and second messengers (Schaefer et al., 1998; Lishko et al., 2011; Strünker et al., 2011; Brenker et al., 2012). Instead, prostaglandins activate CatSper rather *via* an ionotropic mechanism by binding to a subunit or a so far unknown protein associated with the channel complex. Our comprehensive analysis of the structure–activity relationship might enable the development of chemical tools, e.g., prostaglandin photo-affinity labels, to elucidate the prostaglandin-binding site in human sperm as well as the coupling between binding of prostaglandins and channel opening. In contrast to steroids, prostaglandins increase [Ca^2+^]_i_ in sperm with different potency, but rather similar efficacy, suggesting that this class of ligands evokes rather stereotypical behavioral responses in human sperm. This question as well as whether prostaglandins also modulate the activity of Slo3 and whether or not this might co-determine the potency and efficacy of ligands to elevate [Ca^2+^]_i_ in sperm remains, however, to be elucidated. It is also unknown whether only steroid or also prostaglandin activation of CatSper triggers oscillations of [Ca^2+^]_i_. The first encounter of sperm with prostaglandins is in the ejaculate. The physiological function of prostaglandins in the ejaculate is only ill-defined. The hormones might modulate uterine contractions and, thereby, aid the passive transport of sperm from the uterus to the oviduct. In line with Schaefer et al. (1998), we found that in the ejaculate, the action of prostaglandins on CatSper is rather suppressed by Zn^2+^, which might serve to protect sperm from premature ligand-evoked Ca^2+^ influx and ensuing behavioral responses. The mechanism underlying the opposing action of prostaglandins and Zn^2+^ is unknown. Further studies need to address whether Zn^2+^ impairs the binding of prostaglandins, the coupling of binding into channel gating, or both. The Zn^2+^ inhibition of prostaglandin activation of CatSper tapers off upon dilution of the seminal fluid, which might ensure that CatSper is activated only when sperm start escaping from the ejaculate into the female genital tract. In fact, the action of Zn^2+^ in sperm and its role during fertilization are complex and only ill-defined (Allouche-Fitoussi and Breitbart, 2020). It has been reported that seminal Zn^2+^ decreases sperm motility and that the motility is stimulated only upon its removal (Allouche-Fitoussi and Breitbart, 2020); in sperm purified from the ejaculate, stimulation with low micromolar Zn^2+^ concentrations evoked hyperactivated motility (Allouche-Fitoussi et al., 2018; Allouche-Fitoussi and Breitbart, 2020). Our results suggest an even more complex control of motility by a Zn^2+^/prostaglandin interplay, which needs to be scrutinized in future studies that investigate the motility responses of sperm upon dilution from the ejaculate and/or upon stimulation with dilutions of Zn^2+^/prostaglandin mixtures. Moreover, it needs to be investigated whether Zn^2+^ also inhibits CatSper in species, in which the channel is insensitive to hormone activation [e.g., mouse (Lishko et al., 2011)], and whether and how relief of CatSper from Zn^2+^ inhibition affects the swimming behavior of sperm in these species. Prostaglandin activation of CatSper by dilute seminal fluid should rest predominantly on the action of PGE_1_, considering the high micromolar concentration of PGE_1_ in the ejaculate and its low nanomolar EC_50_ (Supplementary Table 3). Similarly, PGEs seem to be the most abundant prostaglandins in oviductal fluid and the secretion of cumulus cells, suggesting that prostaglandin activation of CatSper in the oviduct and close to the egg is largely mediated by PGEs. The concentration of prostaglandins in oviductal fluid is in the nanomolar range (Ogra et al., 1974), whereas their concentration in the cumulus cell secretions is unknown. Of note, both in the oviduct and close to the egg, sperm encounter prostaglandins and steroids at the same time (Schuetz and Dubin, 1981). Prostaglandins and steroids activate CatSper in a strongly synergistic fashion and, thereby, elevate [Ca^2+^]_i_ to levels that are not reached by each ligand alone (Brenker et al., 2018a). Future studies have to take this synergism into account: it remains to be elucidated how the combined action of steroids and prostaglandins affects human sperm functions, such as the swimming behavior and acrosomal exocytosis, under conditions that experimentally emulate the complex chemical, topographical, and hydrodynamic landscapes of the female genital tract. This might provide further insight into the ligand control of sperm behavior during fertilization.

In a nutshell, the results presented here underscore that fertilization is orchestrated by a complex action of steroids and prostaglandins, controlling the activity of CatSper. The precise role of these hormones during fertilization has, however, not been definitely established (Baldi et al., 2009; Tamburrino et al., 2020). We propose that at the beginning of their journey across the genital tract, i.e., in the vagina and uterus, sperm are exposed primarily to high concentrations of prostaglandins rather than steroids acting on CatSper. In the oviduct, yet, still rather afar from the egg, low concentrations of both hormones might act in concert to control the activity of CatSper, whereas at the site of fertilization, steroids present at high concentrations rather than prostaglandins take center stage as to the control of CatSper and, thereby, the function of sperm (Supplementary Figure 5).

## Materials and Methods

### Semen Samples

Semen samples were obtained from normozoospermic (according to WHO) volunteers (donors) and *CATSPER2^–/–^* patients (see Schiffer et al., 2020 for more details) with prior written consent and under approval from the ethical committees of the medical association Westfalen-Lippe and the medical faculty of the University of Münster (reference number: 4INie). Semen samples were produced by masturbation and ejaculated into plastic containers. The samples were allowed to liquefy for 15∼30 min at 37°C, and motile sperm were purified by a swim-up procedure. Of note, performing the swim-up procedure with ejaculates from *CATSPER2^–/–^* patients yields morphologically normal sperm displaying rather normal basal motility (see also Supplementary Movie EV9 in Schiffer et al., 2020). For swim-up, liquefied semen (0.5–1 ml) was directly layered in a 50-ml Falcon tube below 4 ml of human tubal fluid (HTF) medium, containing (in mM) the following: 97.8 NaCl, 4.69 KCl, 0.2 MgSO_4_, 0.37 KH_2_PO_4_, 2.04 CaCl_2_, 0.33 Na-pyruvate, 21.4 lactic acid, 4 NaHCO_3_, 2.78 glucose, and 21 HEPES, pH 7.35 (adjusted with NaOH). Alternatively, the liquefied semen was diluted 1:10 with HTF, and sperm, somatic cells, and cell debris were pelleted by centrifugation at 700 × *g* for 20 min (37°C). The pellet was resuspended in the same volume HTF, 50 ml Falcon tubes were filled with 5 ml of the suspension, and cells were pelleted by centrifugation at 700 × *g* for 5 min (37°C). In both cases, motile sperm were allowed to swim up into HTF for 60–90 min at 37°C. Sperm were washed two times by centrifugation at 700 × *g* for 20 min at 37°C and resuspended in HTF at a density of 1 × 10^7^ sperm/ml. Except for the experiments investigating the action of Zn^2+^, the HTF was fortified with human serum albumin (3 mg/ml) (HTF^+^).

### Measurement of Changes in [Ca^2+^]_i_ and pH_i_

Experiments with swim-up sperm from donors and *CATSPER2^–/–^* patients were performed under exactly the same experimental conditions, ensuring the direct comparability of results. Changes in [Ca^2+^]_i_ were measured in 384 multiwell plates in a fluorescence plate reader (FLUOstar Omega, BMG Labtech, Ortenberg, Germany) at 30°C as previously described (Schiffer et al., 2014). To investigate the action of Zn^2+^, sperm in HTF were loaded with the fluorescent Ca^2+^ indicator Fluo-4-AM (5 μM) for 45 min at 37°C. For all the other experiments, sperm in HTF^+^ were loaded with Fluo-4-AM for 20 min at 37°C. After incubation, excess dye was removed by centrifugation (700 × *g*, 5 min, room temperature) and sperm were resuspended in HTF at a concentration of 5 × 10^6^/ml. Each well was filled with 50 μl of the sperm suspension. Fluorescence was excited at 485 nm and emission was recorded at 520 nm with bottom optics. Changes in Fluo-4 fluorescence are depicted as Δ*F*/*F* (%), i.e., the change in fluorescence (Δ*F*) relative to the mean basal fluorescence (*F*) before application of buffer or stimuli (25 μl), correcting for intra- and interexperimental variations in basal fluorescence among individual wells. Stimuli and buffer were injected into the wells simultaneously using a multichannel pipette. Each condition was measured in duplicates and the two fluorescence traces were averaged. If feasible, the signal amplitudes were fitted (nonlinear, least squares) using a modified Hill equation to determine the EC_50_ value. Changes in pH_i_ were measured in sperm loaded in HTF^+^ with the pH indicator BCECF (5 μM) for 20 min at 37°C. After loading, cells were washed by centrifugation at 700 × *g* for 5 min at 37°C and resuspended in HTF at a concentration of 2 × 10^7^/ml. Fluorescence was alternatingly excited with 440 and 485 nm, and the emission was recorded at 510 nm. Changes in the fluorescence ratio (440_ex_510_em_/485_ex_510_em_) are depicted as Δ*R*/*R* (%), i.e., the change in fluorescence ratio (Δ*R*) relative to the mean basal fluorescence ratio (*R*) before application of buffer or stimuli (25 μl).

### Collection and Handling of Follicular Fluid Samples

Samples were collected from four normoovulatory women (age 30–32, BMI ≤ 25 kg/m^2^, no diagnosis of polycystic ovary syndrome) undergoing ICSI (intracytoplasmatic sperm injection) after ovarian gonadotropin stimulation at the University Hospital in Münster with prior written informed consent under approval from the ethical committees of the medical association Westfalen-Lippe and the medical faculty of the University of Münster (reference number: 1IX Greb 1). All women were treated with 10–14 days of GnRH agonist alone followed by adding a variable dose of 100–300 IU FSH to achieve a controlled ovarian hyperstimulation. Follicular fluid was collected only from large follicles (16–20 mm) 35–36 h after application of 6,500 IU recombinant hCG (Ovitrelle, Merck-Serono, Geneva, Switzerland). Follicular fluid was aspirated using a trans-vaginal single lumen follicle aspiration needle (Gynetics, Gynemed, Lensahn, Germany) filled with 1 ml flushing medium (Gynemed) under ultrasonographic guidance. The follicular fluid from multiple follicles from each patient was pooled and transferred to a 50-ml Falcon tube and placed on ice. After collection, the pooled samples were centrifuged at 1,500 × *g* for 15 min at 4°C to remove cells and debris. The collected supernatant was aliquoted and stored at –20°C.

For comparison, samples were collected from 10 normoovulatory women (age 38–42) undergoing ICSI without ovarian gonadotropin stimulation (natural cycle ICSI) due to male infertility at the University Hospital in Bern with prior written informed consent under approval from the ethical committees of the cantonal ethical committee of Bern, Switzerland (reference: 2020-01682). Serum luteinizing hormone (LH) and 17β-estradiol (E2) levels were monitored throughout the cycle until the diameter of the follicle reached at least 18 mm. At this stage, the E2 concentration was generally above 800 pmol/l, and 5,000 IU of hCG (Predalon^®^, MSD Merck Sharp & Dohme GmbH, Lucerne, Switzerland) was administered 36 h prior to oocyte retrieval. Follicle aspiration for oocyte retrieval was performed without anesthesia. The obtained single follicle fluids were clarified by two-step centrifugation at 600 × *g* for 10 min and then at 1,300 × *g* for a further 10 min. The obtained supernatants were stored at –80°C until the analyses of hormones were performed.

### Collection and Handling of Human Seminal Fluid

After liquefaction, 100 μl of the ejaculate was diluted with 700 μl HTF. The sample was centrifuged (700 × *g*, 20 min, room temperature) to pellet cells and debris. The supernatant was collected and evaluated for the presence of sperm. In case of cellular contamination, the centrifugation step and supernatant collection was repeated until no more sperm were present. The seminal fluid was aliquoted and stored at –20°C.

### Preparation of Prostaglandin Stock Solutions

Prostaglandins were obtained from Cayman Chemical (Ann Arbor, MI, United States). Most of the prostaglandins were obtained as lyophilized powder, from which stock solutions of 20 mM in DMSO were prepared. The stock solutions were aliquoted and stored at –20°C. In case prostaglandins were delivered as stock solutions in a solvent different from DMSO, the solvent was evaporated under a stream of nitrogen and the solute was directly resuspended in DMSO to yield a 20-mM stock solution that was aliquoted and stored at –20°C.

### Preparation of Steroid Stock Solutions

Steroids were obtained as powder from the companies indicated below. The steroids were dissolved in DMSO at a concentration of 20 mM, aliquoted, and stored at –20°C.

**Table:** 

Steroid	Company	Steroid	Company
Pregnenolone	Sigma	DHEA	Sigma
Progesterone	Sigma	Androstenedione	Sigma
Deoxycorticosterone	Steraldoids	Estrone	Merck
Corticosterone	Sigma	Androstenediol	Sigma
17-OH-pregnenolone	Sigma	Testosterone	Sigma
17-OH-progesterone	Sigma	Estradiol	Sigma
11-Deoxycortisol	Merck	PGE_1_	Cayman Chemical
Cortisol	Sigma	DHT	Sigma

### LC-MS/MS Methods, Reagents, and Calibrators—Analyzing the Steroid Profile of FF

The steroid hormones were determined by LC-MS/MS as described (Kulle et al., 2010, 2013; Reinehr et al., 2020). In brief, aliquots of FF samples, calibrator, and controls with a volume of 0.05 ml were combined with the internal standard mixture to monitor recovery. All samples were extracted using Oasis MAX SPE system plates (Waters, Milford, MA, United States). Extracts of estrogens were reduced to dryness under oxygen-free nitrogen (40°C) and the residues were derivatized with dansyl chloride. LC-MS/MS was performed using a Waters Quattro Premier/XE triple-quadrupole mass spectrometer connected to a Waters Acquity (Waters). The chromatographic separation was carried out using an UPLC system, which is connected to a Quattro Premier/XE triple Quad mass spectrometer (Waters). A Waters Acquity UPLC BEH C18 column (1.7 μm, 100 × 2.1) was used at a flow rate of 0.4 ml/min at 50°C. Water (A) and acetonitrile (B) with 0.01% formic acid were used as mobile phase. Two mass transitions were monitored. The following optimized voltages were used: capillary voltage 3.5 kV, cone voltage 28–33 V, collision energy 18–25 eV, dwell time 0.01–0.08 s depending on the steroid, source temperature 120°C, and desolvation temperature 450°C. Argon was used as collision gas. Data were acquired with MassLynx 4.1 software and quantification was performed by QuanLynx software (Waters). During all the analyses, the ambient temperature was kept at 21°C by air conditioning.

### Quantification of the Follicular Fluid Dilution Factor by LC-MS

Follicular fluid is diluted during the collection with washing medium, which contains the synthetic pH buffer HEPES. To determine the dilution factor of follicular fluid, we quantified the concentration of HEPES both in the washing medium and in the follicular fluid. The HEPES concentration in follicular fluid was determined by quenching with acetonitrile/water (1:1) in a ratio of 1:3 for protein precipitation [solvents were purchased in LC-MS grade: water, formic acid (VWR Chemicals, Langenfeld, Germany) and acetonitrile (Thermo Fisher Scientific, Schwerte, Germany)]. After storing in a water/ice bath (10 min, 0°C), the suspension was centrifuged (10 min, 4°C, 16,000 rpm) and the supernatant was filtrated using a syringe filter (H-PTFE, 0.2 μm pore size, 13 mm membrane diameter, Macherey-Nagel, Düren, Germany). The filtrate was diluted with water (3:50) and different concentrations of HEPES were added as a standard to aliquots of the solution. To determine the HEPES concentration in the washing medium, it was filtrated using a syringe filter. The filtrate was diluted with water (1:100) and different HEPES concentrations were added as a standard. Samples were prepared in triplicates and analyzed with the LC-MS method (Borgel et al., 2019). Peaks for HEPES were integrated using the extracted ion chromatograms ([M-H]^–^ with 237.0915 ± 0.01 Da), and the HEPES concentrations in follicular fluid and washing medium were interpolated. Finally, the steroid concentrations determined in follicular fluid were corrected for the dilution with washing medium, yielding the true steroid concentration in crude follicular fluid. HPLC-DAD: solvent rack (SRD 3600); pump (DGP-3600RS); autosampler (WPS-3000RS); column oven (TCC-3000RS); precolumn: Phenomenex security guard TM cartridge C18 (4.0 × 2.0 mm, 4.0 μm); column: Phenomenex Synergi Hydro RP (50 × 2.1 mm, 2.6 μm); temperature 30°C and DAD-detector (DAD-3000RS). The LC system was coupled with a micrOTOF-Q II (Bruker Daltonics, Bremen, Germany). The ESI-qTOF was operated in negative ion polarity in the full scan mode (*m*/*z* = 70–700) with the following settings: capillary voltage 4,500 V, end plate offset -500 V, collision cell RF 300.0 Vpp, nebulizer 2.0 bar, dry heater 200°C, and dry gas 9.0 L/min. Data handling and control of the system were realized with the software Data Analysis and Hystar from Bruker Daltonics. The calibration of the TOF spectra was achieved by injection of LiHCO_2_ (isopropanol/bidist. water 1:1, 10 mM) *via* a 20-μl sample loop within each LC run at 9.0–9.2 min. LC parameters: solvent A: water/acetonitrile 90:10 + 0.1% HCO_2_H; solvent B: acetonitrile/water 90:10 + 0.1% HCO_2_H; pump 1: flow rate: 0–7 min: 0.4 ml/min, 7–9.9 min: 0.6 ml/min, 9.9–10.0 min: 0.4 ml/min; gradient elution: (A %): 0–5 min: gradient from 100 to 0%, 5–6.5 min: 0%, 6.5–7 min: gradient from 0 to 100%, 7–10 min: 100% (v/v).

### Data Analysis and Statistics

Data were analyzed using Microsoft Excel, GraphPad Prism 5, and OriginPro 2020 (OriginLab, Northampton, MA, United States). Data are shown as mean ± standard deviation (SD) with “*n*” referring to the number of independent experiments performed with sperm samples from at least two different donors or *CATSPER2^–/–^* patients. We did not perform tests for statistical significance, because we identified no set of data where this would have been meaningful and/or required to scrutinize the conclusion(s) drawn from it.

## Data Availability Statement

The original contributions presented in the study are included in the article/Supplementary Material, further inquiries can be directed to the corresponding authors.

## Ethics Statement

The studies involving human participants were reviewed and approved by Ethical Committees of the Medical Association Westfalen-Lippe and the Medical Faculty of the University of Münster (reference number: 4INie and 1IX Greb 1) and ethical committees of the Cantonal Ethical Committee Bern, Switzerland (reference: 2020-01682). The patients/participants provided their written informed consent to participate in this study.

## Author Contributions

CBr and TSt conceived the project. JJ, CBi, TSc, IW, FB, DS, AS, AK, PH, MW, BW, VN, TSt, and CBr designed the research; performed the experiments, acquired, analyzed, and/or interpreted the data. JJ, TSt, and CBr drafted the manuscript. All authors revised the manuscript critically for important intellectual content and approved the manuscript for publication.

## Conflict of Interest

Since this study, author AS was employed by MVZ Regensburg BmbH; this company was not involved in the study or its design. The authors declare that the research was conducted in the absence of any commercial or financial relationships that could be construed as a potential conflict of interest.

## Publisher’s Note

All claims expressed in this article are solely those of the authors and do not necessarily represent those of their affiliated organizations, or those of the publisher, the editors and the reviewers. Any product that may be evaluated in this article, or claim that may be made by its manufacturer, is not guaranteed or endorsed by the publisher.
