# Supplementary material for: The Action of Reproductive Fluids and Contained Steroids, Prostaglandins, and Zn2+ on CatSper Ca2+ Channels in Human Sperm
Source: Front Cell Dev Biol. 2021 Jul 26;9:699554. doi: 10.3389/fcell.2021.699554 (PMC8350739; doi:10.3389/fcell.2021.699554)
Supplement: Supplementary file 1 [file Data_Sheet_1.pdf]

Supplementary data

Table S1: Comparison of the steroid composition of follicular fluid from women undergoing different hormonal stimulation.

Concentrations of steroids found in FF (nmol/L)		
**Hormones**	**FF (with gonadotropin stimulation)**	**FF (without gonadotropin stimulation)**
**Progesteron**	28,880	43,793
**Pregnenolone**	1,470	1,056
**17-OH-Progesteron**	2,982	5,813
**17-OH-Pregnenolone**	115	113
**11-Deoxycorticosterone**	65	43
**Corticosteron**	6	6
**11-Deoxycortisol**	0.03	6
**21-Deoxycortisol**	0.19	50
**Cortison**	20	51
**Androstendion**	4	30
**Androstenediol**	20	35
**Dehydroepiandrosterone**	463	227
**Testosteron**	2	5
**Estradiol**	1,221	2,719
**Estrone**	76	161
**Estriol**	8	55

Table S2: Concentration of steroids in follicular fluid and the potency and relative efficacy of these steroids to increase $[Ca^{2+}]_i$ in human sperm.

	Conc (nM)	EC50 (nM)	Rel Efficacy
**Progesterone**	28880	14 ± 1 (n=44)	1 (n=10)
**Pregnenolone**	1470	147 ± 46 (n=11)	0.78 ± 0.06 (n=10)
**17-OH-Progesterone**	2982	13 ± 1 (n=4)	0.98 ± 0.05 (n=5)
**17-OH-Pregnenolone**	115	565 ± 25 (n=4)	0.27 ± 0.03 (n=5)
**11-Deoxycorticosterone**	65	605 ± 25 (n=4)	0.84 ± 0.12 (n=5)
**Corticosterone**	6	4,020 ± 2,100 (n=4)	0.53 ± 0.1 (n=5)
**11-Deoxycortisol**	0.03	2,280 ± 1,000 (n=4)	0.68 ± 0.11 (n=5)
**21-Deoxycortisol**	0.19	ND	ND
**Cortisone**	20	ND	ND
**Androstendione**	4	501 ± 222 (n=4)	0.68 ± 0.07 (n=5)
**Androstenediol**	20	1,130 ± 760 (n=6)	0.39 ± 0.09 (n=5)
**Dehydroepiandrosterone**	463	2,000 ± 696 (n=4)	0.68 ± 0.06 (n=5)
**Testosterone**	2	650 ± 387(n=5)	0.75 ± 0.09 (n=5)
**Estradiol**	1221	1,470 ± 407 (n=7)	0.53 ± 0.11 (n=5)
**Estrone**	76	923 ± 887(n=4)	0.58 ± 0.05 (n=5)
**Estriol**	8	ND	ND
**DHT**	ND	295 ± 174 (n=3)	0.91 ± 0.11 (n=5)
**Cortisol**	ND	1,420 241 (n=6)	0.43 ± 0.06 (n=5)

Table S3: Potency and relative efficacy of prostaglandins to increase $[Ca^{2+}]_i$ in human sperm.

	EC50 (nM)	Rel Efficacy
**PGE1**	5.8 ± 1.8 (n=4)	1
**19-OH-PGE1**	952 ± 48 (n=4)	1.03 ± 0.13 (n=4)
**PGD1**	2,278 ± 1,866 (n=3)	1.3 ± 0.03 (n=3)
**PGF1α**	199 ± 74 (n=3)	1.02 ± 0.02 (n=3)
**PGH1**	202 ± 48 (n=3)	1.04 ± 0.03 (n=3)
**PGE2**	335 ± 110 (n=3)	1.01 0.05 (n=3)
**19-OH-PGE2**	4,892 ± 2256 (n=3)	0.93 ± 0.09 (n=3)
**PGH2**	3,986 ± 2837 (n=3)	0.93 ± 0.04 (n=3)
**AA**	4,190 ± 5147 (n=4)	0.77 ± 0.08 (n=4)
**DGLA**	8,232 ± 2154 (n=3)	0.73 ± 0.07 (n=3)

Supplementary Figures:

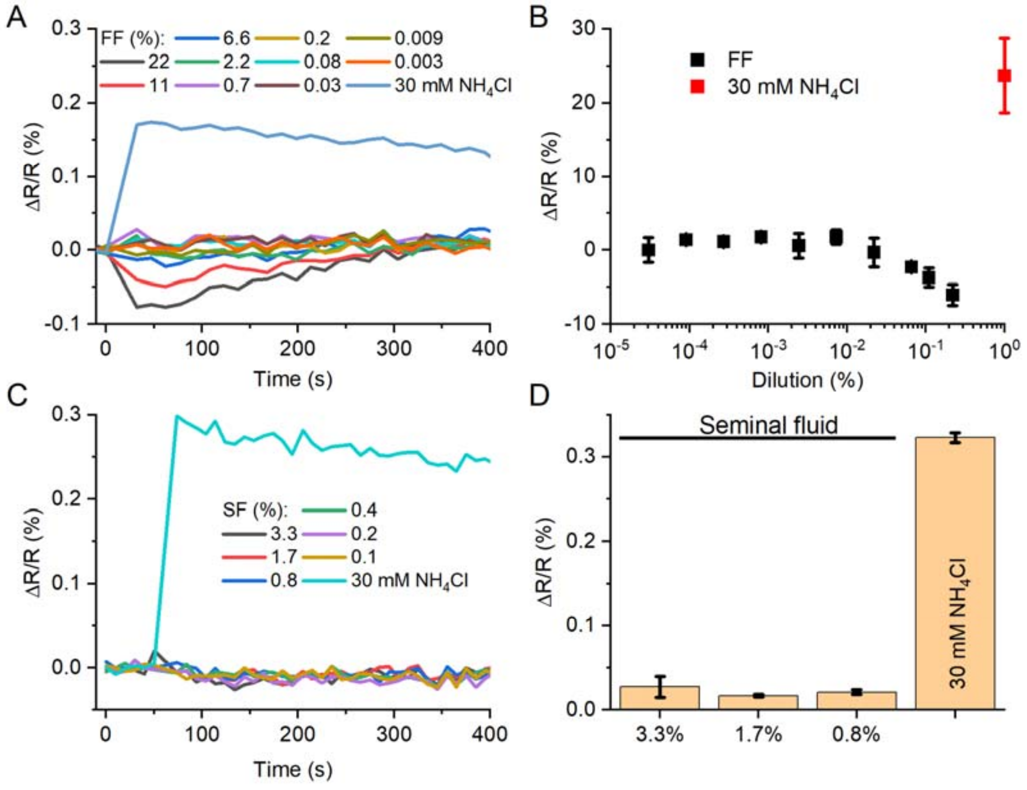

Supplementary Figure 1: Follicular and seminal fluid-induced changes in the pH_i of human sperm (A) FF-evoked pH signals in human sperm. pH_i was monitored in control sperm from healthy donors loaded with a fluorescent pH indicator using a fluorescence plate reader. ΔR/R (%) indicates the percent change in fluorescence ratio (ΔR; R = $F440_{ex}/F480_{ex}$) with respect to the mean basal fluorescence ratio (R) before application (and subsequent continuous presence) of FF at t = 0. NH_4Cl-induced increase in pH_i served as a positive control. **(B)** Mean (± SD) maximal amplitude of FF-evoked changes in pH_i of human sperm. **(C)** SF-evoked pH signals in human sperm. **(D)** Mean (± SD) maximal amplitude of SF-evoked changes in pH of human sperm.

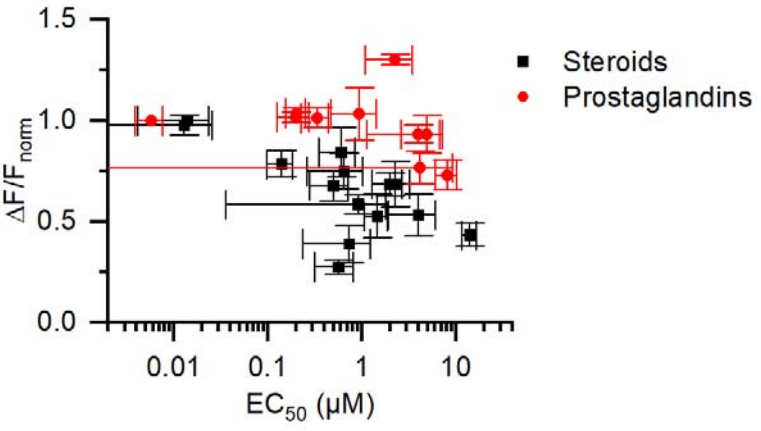

Supplementary Figure 2: Relation between potency and efficacy of prostaglandins and steroids to evoke Ca^{2+} signals in human sperm. Plot of the potency (x-axis) vs. relative efficacy (y-axis) for steroid- and prostaglandin-evoked Ca^{2+} signals in human sperm. Steroid- and prostaglandin-evoked Ca^{2+} signals were normalized to a Ca^{2+} signals evoked by saturating concentration of progesterone and PGE_1, respectively.

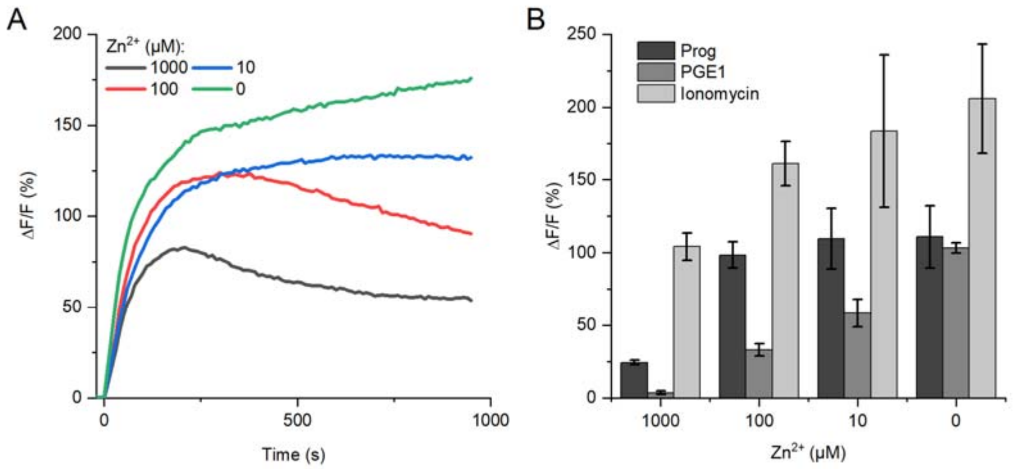

Supplementary Figure 3: (A) Representative ionomycin-evoked Ca^{2+} signals in human sperm in the absence and presence of Zn^{2+}. **(B)** Mean (± SD) maximal amplitude of Ca^{2+} signals in the absence and presence of Zn^{2+} (n=3).

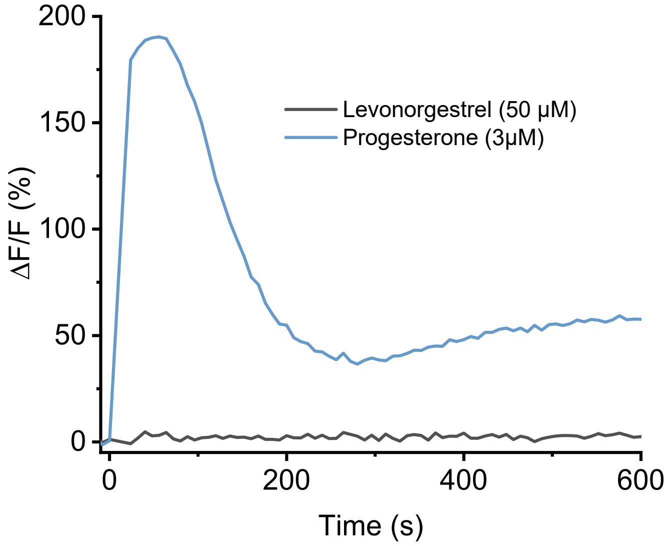

Supplementary Figure 4: The action of Levonorgestrel on CatSper in human sperm. Ca^{2+} signals in human sperm evoked by Levonorgestrel (50 µM) or progesterone (3 µM).

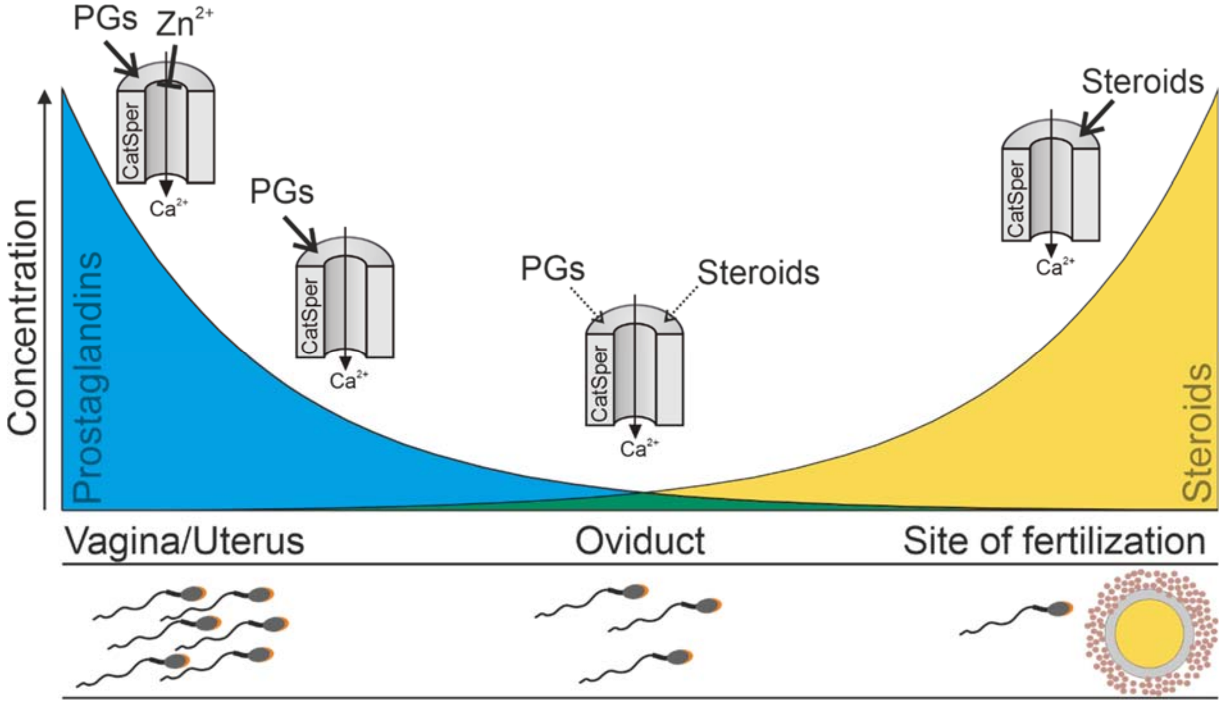

Supplementary Figure 5: Activation of CatSper by steroids and prostaglandins across the female genital tract – a model. In the vagina and uterus, sperm are exposed primarily to high concentrations of prostaglandins rather than steroids acting on CatSper. In the oviduct, low concentrations of both hormones might act in concert to control the activity of CatSper, whereas at the site of fertilization, steroids are present at high concentrations and control CatSper and, thereby, the function of sperm.
